# Parents’ Experience and Psychoeducation Needs When Supporting a Young Person Who Self-Harms

**DOI:** 10.3390/ijerph17103662

**Published:** 2020-05-22

**Authors:** Karolina Krysinska, Sophie Curtis, Michelle Lamblin, Nina Stefanac, Kerry Gibson, Sadhbh Byrne, Pinar Thorn, Simon M. Rice, Alison McRoberts, Anne Ferrey, Yael Perry, Ashleigh Lin, Sarah Hetrick, Keith Hawton, Jo Robinson

**Affiliations:** 1 Orygen, Parkville, VIC 3052, Australia; michelle.lamblin@orygen.org.au (M.L.); nstefanac1@gmail.com (N.S.); sadhbh.byrne@orygen.org.au (S.B.); pinar.thorn@orygen.org.au (P.T.); simon.rice@orygen.org.au (S.M.R.); alison.mcroberts@orygen.org.au (A.M.); s.hetrick@auckland.ac.nz (S.H.); jo.robinson@orygen.org.au (J.R.); 2 Centre for Youth Mental Health, The University of Melbourne, Parkville, VIC 3052, Australia; 3 North Western Mental Health, Parkville, VIC 3050, Australia; sophie.curtis@mh.org.au; 4 School of Psychology, The University of Auckland, Auckland 1142, New Zealand; kl.gibson@auckland.ac.nz; 5 Nuffield Department of Primary Care Health Sciences, University of Oxford, Oxford OX2 6GG, UK; anne.ferrey@phc.ox.ac.uk; 6 Telethon Kids Institute, University of Western Australia, Nedlands, WA 6009, Australia; yael.perry@telethonkids.org.au (Y.P.); Ashleigh.Lin@telethonkids.org.au (A.L.); 7 Department of Psychological Medicine, The University of Auckland, Auckland 1142, New Zealand; 8 Centre for Suicide Research, University of Oxford, Oxford OX3 7JX, UK; keith.hawton@psych.ox.ac.uk; 9 Oxford Health NHS Foundation Trust, Oxford OX3 7JX, UK

**Keywords:** parents, carers, self-harm, young people, support, psychoeducation

## Abstract

Background: Self-harm in young people can have a substantial negative impact on the well-being and functioning of parents and other carers. The “Coping with Self-Harm” booklet was originally developed in the UK as a resource for parents and carers of young people who self-harm, and an adaptation study of this resource was conducted in Australia. This paper presents qualitative analysis of interviews with parents about their experiences and psychoeducational needs when supporting a young person who engages in self harm. Methods: The qualitative study drew on semi-structured individual and group interviews with parents (*n* = 19 participants) of young people who self-harm. Data were analysed using Thematic Analysis. Results: The analysis identified six themes: (1) the discovery of self-harm, (2) challenges in the parent-young person relationship, (3) parents’ need to understand self-harm, (4) parents’ emotional reactions to self-harm, (5) the importance of self-care and help-seeking among parents, and (6) the need for psychoeducational resources. Conclusion: The study highlights the need for support for parents and carers of young people who engage in self-harm, including development and adaptation of resources, such as the “Coping with Self-Harm” booklet, of which an Australian version has now been developed.

## 1. Introduction

Self-harm refers to intentional self-poisoning or self-injury, carried out by an individual irrespective of motivation or the extent of suicidal intent [1,2]. Adolescence is the typical period of onset for self-harming behaviour [3,4]. Self-harm may be linked to significant biological and neurological changes taking place during this period, as well as social and cultural stressors of adolescence, such as increased school and work pressure, and involvement in romantic and sexual relationships [5]. Adolescence is also a period of susceptibility to the first onset of mental ill-health and substance misuse [6]. There is variation in international rates of self-harm in adolescents; a meta-analysis of community-based studies conducted in 18 countries reported that the overall lifetime prevalence of self-harm is 16.9%, with rates ranging from 4.1 to 39.3% [7]. Self-harming behaviour is linked to a heightened risk of death by suicide [8,9,10]; however, not all individuals who engage in self-harm have suicidal intent [11]. Self-harm may occur in response to intense emotions and/or psychological distress, including overwhelming negative feelings and hopelessness, and can be a form of coping with distress [12]. Self-harming behaviour is strongly associated with mental ill-health, such as depression, anxiety, and substance misuse, as well as poor educational and occupational outcomes in early adulthood [13].

In addition to negative outcomes for the young person, self-harm can have a substantial impact on the well-being and functioning of parents and other carers [14]. Parents of young people who self-harm frequently report guilt and shame, fear of exacerbating self-harming behaviour, and changed perceptions of the future for themselves and the young people they care for [15,16]. The discovery that their child is self-harming can be unexpected and highly distressing for parents, and can result in losing confidence in one’s own parenting skills, as well as changes in parenting roles and strategies [17]. Parents report difficulties in communication with their young person about self-harm, feel uncertain about how to address the topic [18], and often doubt their ability to support the young person [19]. This is frequently accompanied by a perceived lack of social support and/or information about how to support the young person (i.e., psychoeducation) [20]. Moreover, stigma associated with self-harm may deepen the sense of isolation and prevent parents and other carers from seeking much-needed information and support [21].

In order to address the needs of parents and other carers of young people who self-harm, researchers at the University of Oxford (Oxford, UK) developed a resource titled “Coping with Self-Harm: A guide for parents and carers” [21,22]. This resource is a 12-page brochure aimed at parents and other carers of young people who are self-harming. The booklet is based on research, including qualitative research conducted with parents of young people who self-harmed [Note 1]. The resource provides information about self-harm, its causes and effects, and includes quotes from parents with advice for other parents. The latter is of particular importance, as parents frequently highlight the perceived benefits of sharing and learning from others in a similar situation [21]. The booklet also includes information on first aid for self-harm, self-care for parents, and provides contact details of services and other sources for psychosocial support for parents and their young people.

While studies on the experience and needs of parents who are supporting a young person who self-harms have been conducted in countries such as the UK [17,18,19,20], there is a paucity of such research in Australia. Also, given the high prevalence of self-harm in young people in Australia, there was a need to develop an Australian resource for parents and carers of young people who self-harm [2,4]. This study was designed to address this gap. It involved a collaboration between Orygen in Melbourne, Australia, and the Centre for Suicide Research at the University of Oxford, UK, which allowed us to review and adapt the UK resource for use in Australian youth mental health, community, and educational settings. The study had two aims. The first was to revise and adapt the UK resource, and the Australian version has recently become available [Note 2]. The second was to explore the experiences of participants, i.e., parents of a young person who self-harms. More specifically, this part of the study explored the following research question: how do parents experience supporting a young person who self-harms, and what are their psychoeducational needs? This paper reports the data related to the second aim of the study.

## 2. Materials and Methods

### 2.1. Sample and Recruitment

We recruited parents with experience of supporting a young person (age range 12–25 years) who had engaged in self-harm (either currently or recently). Parents were recruited at two sites that participated in the Australian adaptation study of the “Coping with Self-Harm” resource: Melbourne, Victoria and Perth, Western Australia. In Melbourne, all participants were recruited through one local council and via two headspace centres [Note 3]. In Perth, participants were recruited through one headspace site and from a parent and carer support group. Recruitment was conducted using a combination of posters/advertisements and social media posts on Twitter. Clinicians at participating sites, i.e., access team members, private allied professionals, enhanced care coordinators, and family workers, could also refer potential participants to the researchers. Potential participants were required to return a signed consent form to the research team. Based on the literature [23,24] and our experience in qualitative research [17,18,20,21], we estimated that 15 to 20 participants would suffice to answer the research question. The study was approved by The University of Melbourne’s Human Research Ethics Committee (Ethics ID: 1749632).

We conducted individual and group semi-structured interviews with 19 participants who did not know one another (sixteen mothers and three fathers; no demographic information was collected from participants). Participants were given a choice about whether to participate in group or individual interviews. Researchers highlight the value of allowing participants to choose how they wish to engage in research on sensitive topics [25]. While individual and group interviews are recognized to elicit somewhat different data, the two methods can provide complementary perspectives on a topic [26]. These interviews were conducted between March and August 2018. Eight parents participated in an individual interview with one researcher (J.R., A.L., S.C. or P.T.) and 11 parents participated in two group interviews, which were conducted by two researchers (combination of S.B., M.L. or N.S.). The interview schedule aimed to elicit participants’ views regarding the original UK version of “Coping with Self-Harm” resource, which they were given in advance of the interview. Participants also provided suggestions for the Australian adaptation, including general feedback regarding the resource, such as the content and format, and skills necessary to provide support to young people who engage in self-harm. These questions prompted further discussion about the experience of having a young person who self-harms, and parents’ needs around this. The semi-structured interviews lasted on average 53 minutes (between 26 and 83 minutes). The audio-recordings were transcribed verbatim by a professional service and anonymised prior to analysis.

### 2.2. Research Team and Reflexivity

This study was led by the Suicide Prevention research group at Orygen (Melbourne, Australia) and the Centre for Suicide Research, University of Oxford (Oxford, UK). Two researchers leading the project (J.R., K.H.) are experts in suicide and self-harm research, and their research interests include involvement of carers in the management of self-harm and suicide prevention in young people. Both centres are renowned for the development of psychosocial resources, as well as online and social media applications in suicide prevention. Senior researchers (J.R., K.G., A.L.) trained and supervised project staff, who conducted the interviews (A.L., S.C., M.L., N.S., S.B., P.T.) and analysed the data (K.K., N.S., M.L.). None of the researchers had a prior relationship with the participants. To safeguard consistency in the analysis the research team met regularly to discuss the progress in recruitment and coding, analysis and interpretation of collected data.

### 2.3. Analysis

We conducted a generic thematic analysis involving a combined inductive and deductive approach [27]. Thematic analysis involves an iterative process for systematically identifying, organising, and offering insight into patterns of meaning (themes) across a data set. Two researchers (K.K. and N.S.) analysed the data independently. First, they familiarised and immersed themselves in the data by reading and re-reading the transcripts and listening to the audio-recordings. The second step involved a systematic analysis of the data through generating initial codes, followed by searching for themes (i.e., collapsing or clustering codes). The next step consisted of reviewing potential themes in the context of the entire dataset. Defining and naming themes was the final step. Themes were discussed with another team member (K.G.), and any discrepancies were resolved through peer conversations with a fourth researcher (M.L.) in an approach that draws from a consensual model of data analysis [25]. We used NVivo 10 software (QSR international, Melbourne, Australia, https://www.qsrinternational.com) in the coding process and for data management.

## 3. Results

The analysis identified six themes relating to how participants experience supporting a young person who self-harms: (1) discovering their child’s self-harm, (2) challenges in the parent-young person relationship, (3) need to understand self-harm, (4) emotional reactions to self-harm, (5) self-care and help-seeking, and (6) need for psychoeducational resources (Table 1).

### 3.1. Discovering Self-Harm: “There’s Life before and after Self-Harm”

Many participants reported that the moment of finding out that their young person was self-harming was a shock. They may have had no idea that self-harm was happening *(“I missed it and I don’t know how (…) that happened”* (Parent 12)) and the moment of discovery became a turning point in their lives. In the words of one of the participants: “*we look at our lives now, there’s our life before this happened and then there’s our life after it happened, and it’s completely different”* (Parent 4). This unexpected discovery led some of the participants to reflect on the idea that self-harm could happen in any family. Consequently, they believed that the wider community should be educated about this risk, for instance using the “Coping with Self-Harm” resource: “*Well, [the resource] is an eye-opener. Something I reckon that all parents should read sometime in their life; if they are having kids, they should definitely read it because it could happen to anyone”* (Parent 8).

Several participants commented that living with and learning about self-harm is a long and ongoing process, involving stages with various needs for psychoeducation. One participant described participants who have just started to learn about and understand self-harm as “first-timers”, and another spoke about the “beginning of the road”. Most participants who took part in this study considered themselves to have gained good knowledge and understanding of self-harm over a period of time: “*I’ve sort of read through all this stuff and gone through the whole process of (…) [I am] pretty much up to speed with what’s happening and what we have to do”* (Parent 8).

Nonetheless, most participants still stressed a general lack of resources and expressed their appreciation of the booklet. One of the participants suggested that mental health services should provide parents with information when self-harm is first discovered: *“if you could just get a little baggy of stuff - they just go, here, look, here’s some information. You’re probably not going to look at it right now because you’re still in that crisis mode, but it’s just sitting there”* (Parent 4). At the same time, absorbing information about self-harm may not be easy, as the topic itself can be overwhelming and can trigger strong emotional reactions:

*“I actually did buy a book on self-harm and I didn’t read it. (…) It was really weird because I wanted to skill up in it but I, at the same time emotionally just resisted it because I felt so overwhelmed and I was so worried that I’d just read all this stuff and just feel overwhelmed”*

(Parent 11)

In brief, the discovery of self-harming behaviour overwhelmed most parents to the extent that it became a turning point in their lives. Parents expressed a need for information and resources to help them better understand and support their child.

### 3.2. Challenges in the Parent-Young Person Relationship: “This Is about Both [Parents and Young People]”

Two points were stressed in relation to having a good relationship with young people in the context of self-harm: a) the importance of striking a balance between being open-minded and understanding, while maintaining boundaries and normalcy in the family, and b) keeping communication between parents and young people open, including talking about self-harm. As one participant said: “*this is about both. It’s about the journey of parents supporting their children and hopefully children being able to reach out to their parents”* (Parent 3).

Participants spoke about attitudes that allowed them to effectively support their young people, including keeping an open mind, being patient, and trying to understand self-harm from the young person’s point of view. Nonetheless, these attitudes may clash with the need to maintain clear boundaries and normalcy in the family. One of the participants expressed this dilemma in the following words: “*It’s really just finding that balance. You want them to trust you and that you’ve got their back. You want to be their friend, but you’ve also got to maintain the… parent thing”* (Parent 1). Some participants indicated that an attitude of leniency toward the young person who is engaging in self-harm may be perceived as differential treatment by other siblings.

Participants also raised the need for skilful and appropriate communication with the young person who engages in self-harm. This included “*communicating with [the] child instead of communicating at [them]”* (Parent 5) and being *“prepared to listen to what your child has to say”* (Parent 3). Some participants admitted that they did not know what to say to their young person, and stressed the necessity to remain calm and not yield to panic or anger when faced with self-harm.

Several participants also observed that sometimes a young person may not engage in a conversation; however, it is important to create opportunities for the young person to talk with their parent, to keep trying and not give up. This can be illustrated by the following:

*“There’s lots of stuff that I thought my son wasn’t listening to and now he’s through it he’ll go ‘Mum, do you remember when?’ He’s had me in tears a few times this year, just going, ‘because of you, because you said’ so, yeah, they may not seem like they’re listening but…”*

(Parent 3)

Some participants engaged in open conversations about mental health issues and self-harm with their young person. This included talking about motives for self-harm or assessing current risk: *“if you have an honest relationship with your kid (…) I’m just self-harming, it’s just cutting, it’s just about release, it’s not about - I’m not trying to die”* (Parent 4).

In summary, parents experienced profound challenges in parenting their child who self-harmed. This included finding a balance between empathic listening and providing support, maintaining a sense of normalcy in the family, taking care of other family members, and being aware of their own emotional reactions and attitudes.

### 3.3. Parents’ Need to Understand Self-Harm: “My Biggest Question Is Why”

Many participants emphasised their need to understand self-harm to be able to better support their child who self-harms. They reported having struggled to find answers to questions about motives and reasons for self-harm, as expressed by one of the mothers: “*So, parents do need help and again, why. That’s my biggest question, is why.* (…) *Why, why, why?”* (Parent 7). Some participants commented that their attempts to understand self-harm were hindered by their perception that when they were growing up self-harm *“just didn’t exist”* (Parent 7) and *“[cutting] wasn’t even a noun, it has always been a verb”* (Parent 2). Yet, participants felt that this lack of understanding did not preclude their capability to be empathic and supportive: *“I don’t get it and I don’t have to. (…) I just need to support”* (Parent 4). Participants often felt that even the young people themselves did not know the answer to the question “why self-harm?”, or were not able to articulate this reason.

Participants presented a range of explanations as to why the young person may engage in self-harm. These included anger, an urgent need for emotional release (an *“outlet for pressure”* (Parent 14)), and getting rid of tension (“*Self-harm is generally about now. How you’re feeling right now, to get that feeling to go away now”,* Parent 4). One participant reported: “*I remember having a chat to my boy about self-harming and he actually turned around to me and said ‘Mum do you feel better when you eat chocolate?’ I went, ‘yeah’. He goes, ‘well I feel better when I cut’”* (Parent 3).

Other participants said that young people may experiment with physical pain and push boundaries (“*how far can [a young person] go before it hurts too much?”,* Parent 6), and that there may be a continuum of self-harm ranging “*from exploring to dangerous”* (Parent 2) or a *“lead up to suicide from self-harm”* (Parent 13). Participants suggested that self-harm may *“run in family”* (Parent 7) and be related to upbringing or mental illness. Others wondered whether self-harm might be linked to bullying, or the influences of social media. Some participants could not easily identify any discernible factors. Others recognised that there may be a multitude of reasons why a particular young person engages in self-harm, and acknowledged that all self-harming behaviour should be taken seriously. One mother observed that her two children: “*are totally different. (…) [For] one of them [self-harm] was a release, one of them was an attention seeking, everybody else was doing it, but at the time you have to take it seriously, (…) because what if you’re wrong?*” (Parent 4).

Some participants drew attention to a distinction between self-harm and a suicide attempt. One of the participants reported having experienced both: “*I’ve been on both sides of the fence (…) she’s self-harmed, but she’s also tried to suicide as well. There’s a very big point of difference”* (Parent 4). This difference was of great importance as the young person’s suicide attempt would *“just put you in a panic”* (Parent 4). One of the participants was also wondering whether suicide is the *“next step”* after self-harm (Parent 7).

To sum up this theme, understanding ‘why’ the young person self-harms was crucial for parents, and they considered many reasons, including the release of emotions and experimental behaviour. Some parents also considered familial transmission or external factors, such as bullying or social media, and some parents distinguished self-harm from suicidal behaviour, yet perceived a potential continuum between self-harm and suicidality.

### 3.4. Parents’ Emotional Reactions: “You Might Have Strong Emotions Yourself”

Participants emphasised that supporting a young person who engages in self-harm is overwhelming, upsetting, and can easily trigger intense emotional reactions. Many participants spoke about frustration and helplessness about not knowing what to do to prevent new episodes of self-harm. Some participants have tried doing “*everything by the book”* (Parent 6) and everything which was *“recommended by the psychs, the doctors”* (Parent 6); however, self-harm continued. They spoke about not being able to see the *“light at the end of the tunnel”* (Parent 1) and about being *“backed in the corner”* (Parent 7). For some participants the feelings of frustration and hopelessness were intertwined with anger, as expressed by this mother: *“sometimes you are, you’re just angry. You just think, what did I do wrong? Why? What didn’t I do for you? You know? Like why wasn’t I enough?”* (Parent 4). In addition, this feeling of helplessness could be compounded by the young person’s perceived resistance to seeking help.

Many participants struggled with a feeling that they had *“really failed”* as parents. They spoke about self-blame and guilt, as expressed by one of the participants: *“as a mother you feel guilt a lot, whether you’re in the right or in the wrong”* (Parent 2). There was embarrassment and unwillingness to talk about their children with others, as participants felt that their families did not match an image of a *“perfect family”*. One participant observed that families facing the challenges of mental ill-health are not *“Instagram-able”*: *“It’s interesting how a lot of families go quiet when their children are teenagers because life can be a bit shit in between the Instagram pictures”* (Parent 2). Participants were also sensitive to perceived *“finger pointing”* in existing psychoeducational material (*“there are a lot of myths around, bad parenting equals ADHD [Attention-deficit/hyperactivity disorder] or equals self-harm”,* Parent 11).

Another strong reaction was hypervigilance and a strong incessant fear of repeated self-harm and even suicide. This was expressed by one of the mothers: “*Because the fear, it’s always the fear, is she going to go in her room? Is she going to do it? Have I upset her that much that she’s going to keep doing it sort of thing?”* (Parent 7). Some participants took up a *“full time kind of carer job”* (Parent 11) and others had their thoughts focused *“always on the victim [young person]”* (Parent 6). One of the mothers observed a change in her own behaviour in an attempt to prevent her daughter’s repeated self-harm: *“I’m not a control freak, but I’ve become controlling. Because it’s something I can manage then and I don’t have to be as afraid, if I’m in control”* (Parent 12).

Some participants had decided to share with the young person the impact self-harm had on them. They believed that this would allow the young person to see that it is fine to express emotions. One of the participants also suggested that young people should know how self-harm can affect parents:

*“I just want them to see-because it’s not all - it’s about them, it’s not about us but I want them to see how it affects us. To show them that they weren’t going through all this alone (…)”*

(Parent 7)

Importantly, this theme signals that supporting a young person who self-harms evoked strong feelings in parents, especially frustration and helplessness. Parents felt embarrassment towards others and felt that they had failed in their parenting role. In addition, they became hypervigilant toward and afraid of continuing self-harming behaviour.

### 3.5. Self-Care and Help Seeking: “You’ve Got to Help Yourself before You Can Help Your Child”

There was a consensus among participants regarding the importance of *“getting help yourself”* (Parent 7) and *“plugging yourself in and recharging”* (Parent 1). Participants voiced a view that self-care was necessary in order to effectively support their children. One mother spoke about a *“domino effect”* in the family: “*If the parent is stressed, the main caregiver is stressed then how does that impact around other siblings? The child that you’re trying to support, who’s self-harming, are they looking at it like they’re not coping because I’m… It kind of-yeah, dominoes”* (Parent 3).

It was noted that participants should be able to acknowledge their limitations, feel comfortable sharing their feelings, and the need to seek help and support should be normalised. Nonetheless, participants experienced finding this difficult: *“it’s really easy to put yourself on the backburner and not care for yourself as much as you should”* (Parent 4) and *“my needs were my children”* (Parent 12). Some participants felt guilty about recognising their own needs and attending to them, and for some participants, learning self-care *“has been a struggle”* (Parent 1).

Participants spoke about their self-care activities. These included hobbies, such as cooking and crafts, spirituality and alternative healing techniques, breathing exercises, and mindfulness. One of the mothers advised others to *“go sit with a cup of tea out the front of the house, out the door, on your own for five minutes, stick your headphones on with some music and breathe”* (Parent 15). Some participants have been *“taking it day by day”* (Parent 1) and found looking at challenges from different perspectives (*“wearing different hats”,* Parent 3) helpful.

Several participants experienced support from family and friends, although talking to them about self-harm raised questions about confidentiality and exposing the young person to judgement: *“It did help me because it helped [other family members] (…) to understand what [the young person] was going through, but I didn’t want them to judge her. But they’ve been really good. I only shared it with my brothers and sisters”* (Parent 7).

Participants sought help from mental health professionals and services, and advised other parents to *“tell them [professionals] as much as you can”* (Parent 6). Nevertheless, some participants reported negative experiences with services; some felt they were being blamed for their child’s self-harm or experienced services as *“very clinical and (….) they don’t feel very understanding”* (Parent 3).

Many participants stressed the value of talking to other parents of young people who have self-harmed (i.e., other parents with *“lived experience”*). There was a feeling of immediate connection among parents living with a child who self-harms, being *“on the same wavelength”* and *“just getting it”* (Parent 16). In contrast, there was a perception that people without such experience were not able to understand; they may react in ways which are *“minimising and unhelpful”*, such as *“people sort of go, ‘Oh don’t worry, she’s just following a trend’”* (Parent 11). Participants believed that contact with other parents with lived experience of young people who self-harm could offer them a forum to share experiences, get advice, and find a different perspective and better understanding of self-harm:

*“I’d love to hear from other parents and just sit there - I mean, I’d probably sit there for hours listening. How they handled it and how they worked through it, how the kids are going with it now”*

(Parent 6)

In brief, parents unanimously underscored the importance of self-care, though some felt guilty about taking care of their own needs. Parents utilised various individual self-care activities and social and professional support. Peer support from other parents was especially highly valued, as it provided understanding and normalisation of their experiences.

### 3.6. Need for Psychoeducational Resources: “I Wish I Had Something Like This [Booklet] When I Was Going Through It [Self-Harm]”

Participants expressed appreciation of the “Coping with Self-Harm” booklet [20] and supported its adaptation to the Australian youth mental health context. Reflecting their experience of coping with self-harm, all participants voiced a need for resources containing information about what self-harm is, how it is related to suicide, and explains the possible triggers and reasons for this behaviour. They also expressed the need for information on how to provide both psychological and practical support to their young person, some alternatives to self-harm, and available services. Participants valued information on the possible impact self-harm may have on other family members, including other children, as *“you get all consumed over that one sibling and just leave the others to just get by. You have to be reminded that they’re going through it as well”* (Parent 2).

Participants also highlighted a need for suggestions on how to talk with others, including people outside of the family, about self-harm. For instance, one of the participants advised:

*“There needs to be a discussion with the child that’s actually self-harming about why it needs to be discussed and why it needs to be discussed with certain people. (…) It should be a joint decision on who - say, for myself and my son, who those people were going to be”*

(Parent 3)

Participants found quotes from other parents and young people with an experience of self-harm very useful, such as *“something like it [a way of coping with self-harm] really works, it really makes [the young person] feel better*” (Parent 11). Information on self-care and attending to their own needs was also perceived as being of great value, as it *“reminds parents also to get help for themselves, and that there are resources and information included [in a resource] that reminds parents that you need to take care of yourself while also taking care of your child, because (…) a lot of parents would focus only on their child and not realise that their own mental and physical health are declining”* (Parent 5).

Participants stressed the importance of user-friendly and non-clinical language, and a simple layout of the resource (*“make it really plain and simple”* (Parent 4)). In contrast to the original UK resource, Australian parents expressed a preference for the term “youth” or “young person” instead of “child” in the resource.

To summarise this theme, parents appreciated a resource with easy to understand and comprehensive information about self-harm, and advice on how to support and communicate with a young person. Quotes from other parents and information about self-care were also perceived as helpful. Parents preferred the use of non-clinical language and a user-friendly layout.

## 4. Discussion

This study identified six key themes that reflected the experience and needs of parents supporting a young person who self-harms. These ranged from the need to learn about this behaviour and challenges in the parent-young person dynamic, to emotional reactions to self-harm, and the importance of self-care and help-seeking. As reported in previous studies [17,23], discovery of self-harm is usually unexpected and is described by parents as a shock. In addition to the traditional role of a parent, it necessitates assuming a role of the caregiver of a young person who self-harms, although most parents believe they do not have the knowledge and skills to provide appropriate care and support [20,28]. Consistent with other research among family caregivers of young people [29], participants in our study stressed their need for information and resources about self-harm and its management. Reflecting findings of previous studies [28], parents noted a significant gap: such resources often do not exist, they are difficult to find or receive, or they do not meet their needs. Importantly, participants also noted that parents’ psychoeducational needs evolve over time, which suggests the need to provide appropriate and diverse informational support [29,30,31].

Another theme revealed challenges inherent in the parent-young person dynamic in the context of self-harm. One aspect of this was a challenge of striking a balance between open-mindedness and understanding on the one hand, and discipline and normalcy in the family on the other. The tension between the need for autonomy and support is one of the normative processes in a young person’s transition to adulthood [32]. Nonetheless, this process can be exacerbated and complicated by self-harming behaviour. Previous studies noted that self-harm frequently changes the family dynamics and creates uncertainty about effective parental practices [19,33]. Similar to parents in previous studies [15,16], our participants expressed a fear that setting boundaries could trigger another episode of self-harm and observed that siblings may feel neglected or become resentful of differential treatment of the young person who self-harms. These relational challenges reflect the need to equip parents, who are the primary source of help for young people [14,34], with the right support and resources.

As Whitlock et al. [35] and Rissanen et al. [36] have observed, faced with self-harm, some parents develop new communication skills, which created a sense of partnership and closeness between them and the young person who self-harms. This was also found in our study. Some parents reported having conversations about self-harm with their children, while others were uncertain and asked for information on how to do so effectively. According to young people themselves, parent’s ability to “talk and listen” about self-harm is an effective support strategy [14]. In addition, our study participants emphasised another aspect of supportive communication, specifically, remaining calm and non-judgemental even during emotionally-charged situations with young people. This finding reflects the significance of what Kelada et al. called “calm communication” about self-harm [19] (p. 3406).

The need to understand the reasons and motives for self-harm featured strongly in our interviews, which corresponds to findings of previous studies [16,18,28,36,37]. According to Hughes et al. [18], parents’ attempts at meaning-making generally start with an initial reaction of shock and confusion, followed by searching for information (e.g., talking to the young person about self-harm), and resulting in constructing a feasible explanation. Better understanding of self-harm can help parents to provide more effective care and support [14], although some participants in our study indicated that not understanding why the young person was engaging in self-harm did not stop them from providing support, and having access to too much information may be emotionally overwhelming.

Participants in our study offered various possible explanations for why their children might have engaged in self-harm. These ranged from emotional release and an attempt to manage psychological pain, to experimentation and testing boundaries of physical pain. These explanations were consistent with motives and reasons for self-harm reported in other studies with parents [36] and young people [38]. Some parents wondered about the link between the Internet, in particular social media, and self-harming behaviour. This finding underlines the need for further research in this area, and the development and implementation of online self-harm and suicide prevention and awareness campaigns [2,39]. Importantly, parents emphasised the need to understand the difference between self-harm and a suicide attempt (intent to release negative tension/to feel better versus intent to die), and wanted to understand whether self-harm can escalate or evolve into suicidal behaviour. This finding is consistent with other research on the meaning of self-harm for parents [36]. It also underlines carers’ need for accurate and user-friendly psychoeducation on suicide and self-harm, including the use of consistent terminology [31,40,41,42,43].

The current study supported many of the findings of previous research in relation to parents’ emotional reactions to self-harm [15,16,19,28,33,37]. This growing body of research unequivocally shows the extent and complexity of the family burden related to self-harm [44]. As Shah et al. [44] have highlighted caregiving extends beyond the objective burden related to practical issues, such as financial problems and lack of time. It encompasses subjective burden (i.e., psychological reactions) and grief for what has been lost, including the family lifestyle and an idealised view of what being a family means. Some parents in our study decided to talk to the young person about the negative impact that self-harm has had on their own psychosocial functioning. The existing literature suggests that shame and concern about placing emotional burdens on family and friends are major barriers to disclosure of self-harm among young people [45]. Similarly, it is possible that learning that self-harm has a negative impact on their family members may affect a young person’s willingness to share their experience with parents or affect their motivation to seek support. Thus, there is a tension between a parent and/or carer’s need to communicate the effect their young person’s self-harm is having, and the potential implications of this on a young person’s help-seeking behaviour or well-being. It may be important to educate parents and carers on appropriate ways to communicate the difficulties they are experiencing, without placing additional feelings of shame and burdensomeness on their young person.

Self-care and help-seeking was another prominent theme in our study. Self-care activities mentioned by parents included crafts and hobbies, spirituality, mindfulness, and talking to confidants. The study design does not allow any conclusions to be drawn about effectiveness of these coping strategies. However, other research has found that more active and/or positive coping strategies, such as seeking support, are related to better mental health outcomes across diverse populations of carers [44,46,47]. Further, our study highlights the importance of normalising self-care and help-seeking among carers. Study participants observed that supporting the young person becomes a carer’s priority, and engaging in self-care can easily trigger feelings of guilt. The tendency to “put oneself on backburner” has also been reported in previous studies with parents of young people who self-harm [33]. Given the toll of self-harm on families and carers, it is surprising that only few interventions for parents and carers of young people who self-harm have been developed and evaluated [28,48]. This is a significant gap which needs to be addressed. There is evidence from other fields that supportive and psychoeducational programs for carers of people with mental health issues, including young people, can be effective in reducing the perceived burden and distress [49,50,51].

Participants in our study believed that contact with other parents with lived experience of self-harm may provide a forum to share experiences and advice, to find a different perspective, and to better understand self-harm. Other research has also shown the perceived and actual benefit of peer support for parents managing self-harm [17,28,33]. Nonetheless, reliance on peer support in some cases reflected mixed or negative experiences with mental health services or with parents without experience of caring for a young person engaging in self-harm, including encountering a judgemental attitude. This calls for further education and training for professionals and the general population in addressing stigma and negative attitudes towards families living with self-harm and mental health issues, and the development of appropriate peer support programs.

Study participants strongly supported the adaptation of the original UK-based “Coping with Self-Harm” [22] resource to the Australian youth mental health context, and provided feedback regarding the preferred contents, layout, and language. This information has been used in the adaptation process, and currently an Australian version of the booklet is available free of charge in hard copy and online. The resource is being distributed to youth mental health services, schools, and other organisations and services working with young people and their parents and other carers across Australia. Additionally, there is an ongoing evaluation of the resource in terms of its implementation, acceptability and usefulness.

### Limitations and Strengths of the Study

The study was based on a convenience sample of parents (mostly mothers) from two sites in Australia. Future studies may include other family members, such as siblings, and non-family members, such as friends, who may play a different role in supporting young people who self-harm and may have different information or support needs. In future studies, it may also be of value to involve parent dyads, as having a shared understanding and an agreed approach may be useful to parents providing support to their young person [52]. Nevertheless, the study successfully recruited 19 participants, and the interviews yielded rich data which allowed us to answer the research question. This study contributes to the literature on the impact of self-harm on parents and how parents experience their role as support providers to young people who self-harm. Importantly, it provided crucial new information on parents’ needs for support and self-care, as well as their suggestions for provision of psychoeducation.

## 5. Conclusions

The current study highlights the need for the provision of comprehensive psychosocial and educational support for parents of young people who self-harm. When confronted with self-harming behaviour, parents often struggle with their own distress and feel poorly equipped to provide the help the young person needs. Self-harm can also result in changes in the relationship between parents, the young person, and other family members. Parents clearly voiced their need for psychoeducation on self-harm and its management, as well as the need for both peer support and self-care strategies, and strongly supported the adaptation of the “Coping with Self-Harm” booklet [20] for the Australian context. Despite challenges outlined in this paper, parents are a primary source of support for young people and play a critical role in ensuring young people’s wellbeing. It is essential that they are provided with support and information needed to assist their young person. Mental health services and other frontline service providers can play a vital role in providing support to families, including access to psychoeducational resources.

Note 1: Videorecording of interviews with parents, which have informed development of the “Coping the Self-harm” booklet in the UK are available online (https://www.healthtalk.org/self-harm-parents-experiences/overview).

Note 2: The adapted resource and an accompanying video was made available online (www.orygen.org.au/copingwithselfharm) and in hard copy for interested services in Australia in September, 2019.

Note 3: headspace are free and confidential mental health services in Australia for young people aged 12–25 who have concerns about their mental or physical health (https://headspace.org.au).

## Figures and Tables

**Table 1:** Descriptive themes.

Theme	Content
1. Discovering that the young person is self-harming	Shock and surprise Self-harm can happen in any family and there is general need for psychoeducation about self-harm Learning about self-harm is an ongoing process General lack of psychoeducational resources
2. Challenges in the parent-young person relationship	A balance between being open-minded/understanding and boundaries/normalcy in the family Keeping communication open between parents and young people, e.g., need to remain calm and to keep trying to talk to a young person who self-harms
3. The need to understand self-harm	Understanding self-harm may increase ability to support a young person who self-harms Struggle to understand motives and reasons for self-harm Lack of understanding does not preclude ability to support a young person Explanations as to why the young person may engage in self-harm, such as anger, emotional release, experimenting with physical pain, and stress, and recognition that the motives and reasons for self-harm can be complex Difference between self-harm and a suicide attempt
4. Emotional reactions to self-harm	Providing support can be distressing and can trigger intense emotional reactions Frustration, helplessness, anger Perceived failure as a parent, self-blame, guilt Embarrassment, perceived “finger pointing” Sharing with the young person how self-harm can impact parents
5. Self-care and help-seeking	Importance of self-care and help-seeking, and need to normalise seeking help and support Too easy to forget one’s own needs Self-care activities, e.g., hobbies, mindfulness, and coping strategies, e.g., taking a different perspective Support from family and friends Help from mental health professionals and services Importance of peer support
6. The need for psychoeducational resources	Appreciation of the “Coping with Self-Harm” booklet Need for user-friendly and easy to find information resources on self-harm, e.g., what is self-harm, triggers for self-harm, impact on the family, communication, and self-care for parents Usefulness or quotes from other parents and young people with an experience of self-harm
